# Histone deacetylases regulate organ-specific growth in a horned beetle

**DOI:** 10.1186/s13227-024-00223-5

**Published:** 2024-04-05

**Authors:** Yonggang Hu, Jordan R. Crabtree, Anna L. M. Macagno, Armin P. Moczek

**Affiliations:** 1 Department of Biology, Indiana University, 915 East 3rd Street, Bloomington, IN 47405 USA; 2 State Key Laboratory of Resource Insects, Institute of Sericulture and Systems Biology, Southwest University, No.2 Tiansheng Road, Beibei District, Chongqing, 400715 China; 3 Biostatistics Consulting Center, Department of Epidemiology and Biostatistics, School of Public Health Bloomington, Indiana University, 2719 E. 10th Street, Bloomington, IN 47405 USA

**Keywords:** *Onthophagus*, Chromatin, Plasticity, Nutrition

## Abstract

**Background:**

Nutrient availability is among the most widespread means by which environmental variability affects developmental outcomes. Because almost all cells within an individual organism share the same genome, structure-specific growth responses must result from changes in gene regulation. Earlier work suggested that *histone deacetylases* (*HDACs*) may serve as epigenetic regulators linking nutritional conditions to trait-specific development. Here we expand on this work by assessing the function of diverse *HDACs* in the structure-specific growth of both sex-shared and sex-specific traits including evolutionarily novel structures in the horned dung beetle *Onthophagus taurus*.

**Results:**

We identified five *HDAC* members whose downregulation yielded highly variable mortality depending on which *HDAC* member was targeted. We then show that *HDAC1*, *3*, and *4* operate in both a gene- and trait-specific manner in the regulation of nutrition-responsiveness of appendage size and shape. Specifically, *HDAC 1, 3,* or* 4* knockdown diminished wing size similarly while leg development was differentially affected by RNAi targeting *HDAC3* and *HDAC4*. In addition, depletion of *HDAC3* transcript resulted in a more rounded shape of genitalia at the pupal stage and decreased the length of adult aedeagus across all body sizes. Most importantly, we find that *HDAC3* and *HDAC4* pattern the morphology and regulate the scaling of evolutionarily novel head and thoracic horns as a function of nutritional variation.

**Conclusion:**

Collectively, our results suggest that both functional overlap and division of labor among *HDAC* members contribute to morphological diversification of both conventional and recently evolved appendages. More generally, our work raises the possibility that *HDAC*-mediated scaling relationships and their evolution may underpin morphological diversification within and across insect species broadly.

**Supplementary Information:**

The online version contains supplementary material available at 10.1186/s13227-024-00223-5.

## Background

Phenotypic plasticity is the ability of an organism to change its phenotype in response to environmental stimuli [1, 2], a universal phenomenon in the living world. Diverse abiotic (e.g., temperature, photoperiod) and biotic (e.g., conspecific density) factors may influence growth and differentiation [3–5]. Among those, nutrition is one of the most widespread means by which environmental variability affects developmental outcomes. Lack of essential nutrients can slow or arrest development, and sometimes trigger alternative developmental programs, such as the diapause of insects and worms [6]. Nutrition also serves as a major determinant of animal size and shape [7, 8], with poor nutrition generally yielding reduced growth and final adult body size in animals with determinate growth such as insects and mammals. However, different body parts within an individual typically differ in their response to nutrient availability. For instance, brain size in mammals and male genital size in arthropods are relatively nutrition-insensitive [9, 10], whereas secondary sexual traits such as male horns of dung and rhinoceros beetles [11, 12], or mandibles of stag and broad-horned flour beetles [13, 14] are exquisitely sensitive to nutritional variation during development. Such trait-specific scaling relationships therefore contribute in important ways to shape morphological diversity within and among taxa.

Because all cells within an individual organism essentially share the same genome, organ- or structure-specific growth must result from changes in gene regulation. Much recent work has described changes in transcription profiles in response to environmental modifications such as nutrient availability, and has begun to identify key regulators of condition-responsive growth (e.g., *insulin*/*IIS* [15–17], *doublesex* [18], and *hedgehog* [19]). However, how such variation in gene expression is achieved in the first place, and then subsequently transduced into organ-specific growth is much less well understood. Here, we investigate the role of epigenetic modifications in enabling organ-specific growth, with a particular emphasis on histone modifications.

Histone acetylation/deacetylation is crucial in the organization of euchromatin (which enables transcription) and heterochromatin (which inhibits transcription), thereby mediating changes in gene expression. Histone deacetylases (HDACs) are members of an ancient enzyme family that reverses the acetylation of protein substrates. *HDAC*-mediated removal of acetylation from histone tail lysines generally correlates with gene silencing by decreasing the ability of transcription factors to access DNA [20]. In insects, studies have confirmed that *HDACs* play a role in various developmental processes, such as growth [21], metamorphosis [22–24], long-term memory [25], reproduction [26], longevity [27], caste differentiation [28, 29], diapause [30], and immunity [31]. Furthermore, in the broad-horned flour beetle *Gnatocerus cornutus*, *HDAC1* and *HDAC3* differentially participate in the nutrition-dependent growth of wings and male-exaggerated mandibles, suggesting that *HDACs* may serve as epigenetic regulators linking nutritional conditions to trait-specific development [21]. Here, we expand on this work by assessing the function of diverse *HDACs* in the structure-specific growth of both sex-shared and sex-specific traits including evolutionarily novel structures in a horned dung beetle.

Horned dung beetles (genus *Onthophagus*) have emerged as promising model systems to investigate the development and diversification of scaling relationships. Here we employ one such model, the bull-headed dung beetle *O. taurus*, to investigate the function of five different *HDAC* members in the development of four different morphological structures. We selected hind legs as examples of traits that exhibit moderate nutrition-responsiveness and therefore scale roughly isometrically with body size. We also investigated male genitalia because of their relatively muted nutrition response and corresponding hypoallometric scaling. Finally, we assessed thoracic horns and head horns because of their highly sex-specific growth and scaling relationships [32]. Thoracic horns are observed only in the pupal stage of both sexes where they function as molting devices in the shedding of the larval head capsule during the larval–pupal molt, and exhibit exaggerated growth in males [33, 34]. In partial contrast, head horns are found only in male pupae as well as adults and exhibit extreme nutrition-responsive growth resulting in hyper-allometric scaling with body size. Head horns function as weapons in competition between adult males over reproductive access to females. While thoracic horns have recently been identified as partial wing serial homologs [35], head horns lack any obvious homology with other structures and are thus considered evolutionary novelties even by the strictest of definitions [36, 37]. Below we detail our results and discuss them in the light of the developmental regulation of growth and plasticity in horned beetles in particular and insects broadly.

## Results

**Fig. 1:**
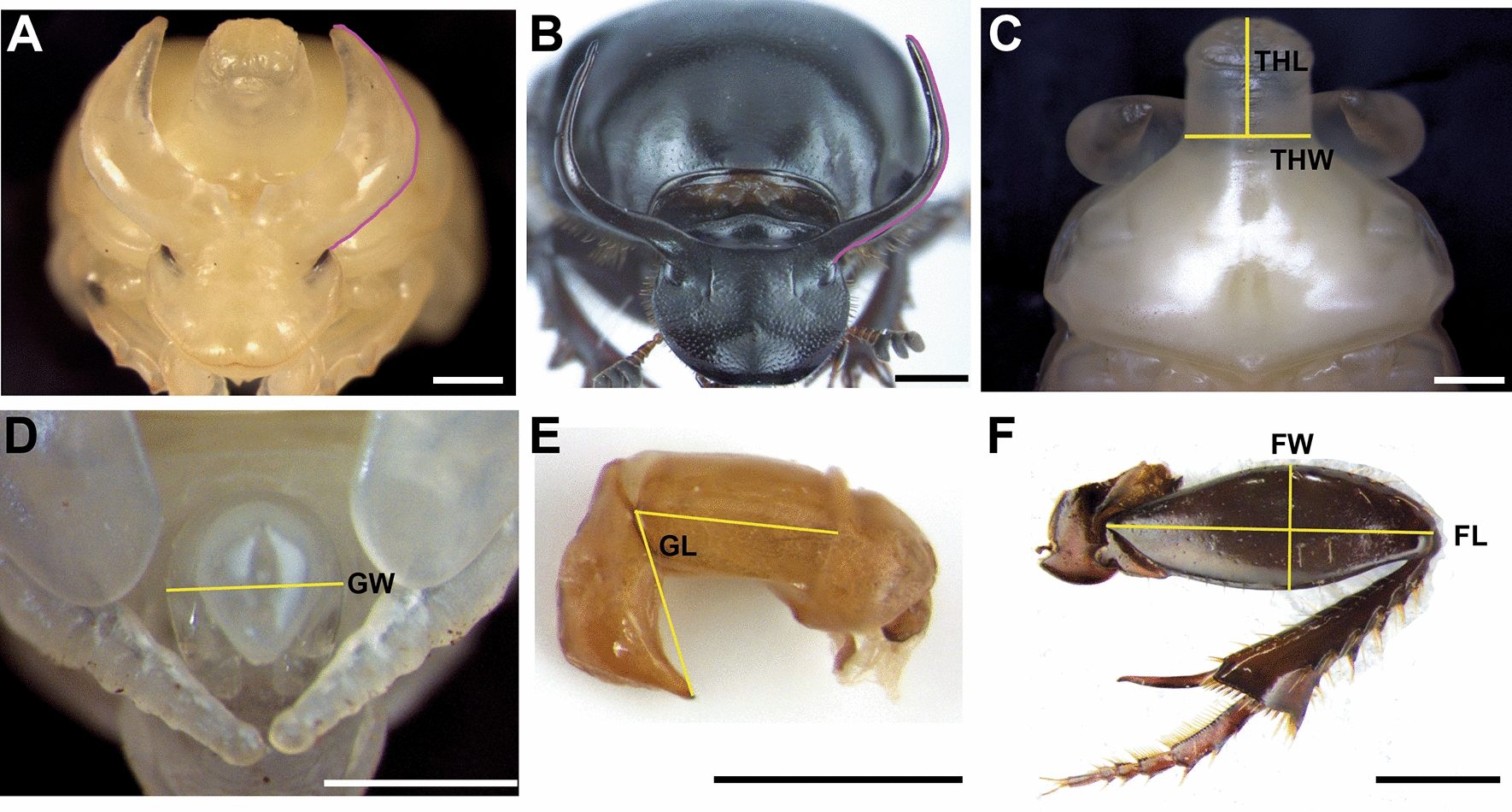
Wildtype morphology and morphometric landmarks used for morphological quantification. **A**–**F** Morphology of the male pupal head (**A**), male adult head (**B**), pupal pronotum from dorsal view (**C**), pupal reproductive organ (**D**), adult aedeagus (**E**), and hind leg (**F**), respectively, and the morphometric landmarks used for measurements (purple and yellow line). *THW* thoracic horn width, *THL* thoracic horn length, *GW* genital width, *GL* genital length, *FL* femur length, *FW* femur width. Scale bars: 1 mm

We sought to characterize the presence and function of *HDACs* during the development of the bull-headed dung beetle *O. taurus*. We identified five HDACs in the annotated genome, which, based on phylogenetic analysis, could be classified into three classes: class I (HDAC1 and HDAC3), class II (HDAC4 and HDAC6), and class IV (HDAC11) (Additional file 1: Fig. S1). It is worth noting that two HDAC proteins that were predicted as HDAC-Rpd3 (reference number: XP_022902140.1) and HDAC5 (reference number: XP_022905538.1) for *O. taurus* in the NCBI database were found to be nested within the cluster containing HDAC1 and HDAC4 proteins (Additional file 1: Fig. S1). As a result, we have re-annotated these two proteins as Ot-HDAC1 and Ot-HDAC4, respectively. We then performed RNAi experiments by injecting double-stranded RNA (dsRNA) corresponding to each of the five HDACs into newly molted final-instar *O. taurus* larvae and assessed their influence on pupal and adult morphologies. Bioinformatic analyses indicated that the maximum number of identical sequences in loci other than the targeted genes did not exceed 11mers for *HDAC1* dsRNA, 8mers for *HDAC3* dsRNA, 14mers for *HDAC4* dsRNA, 16mers for *HDAC6* dsRNA, and 7mers for *HDAC11* dsRNA, respectively, suggesting that off-target effects are an unlikely explanation for the phenotypes documented below though we cannot completely rule out the possibility of off-target effects. Wildtype morphology for each focal phenotype is shown in Fig. 1.

### *HDAC*-RNAi resulted in highly variable mortality depending on the target *HDAC*

**Fig. 2:**
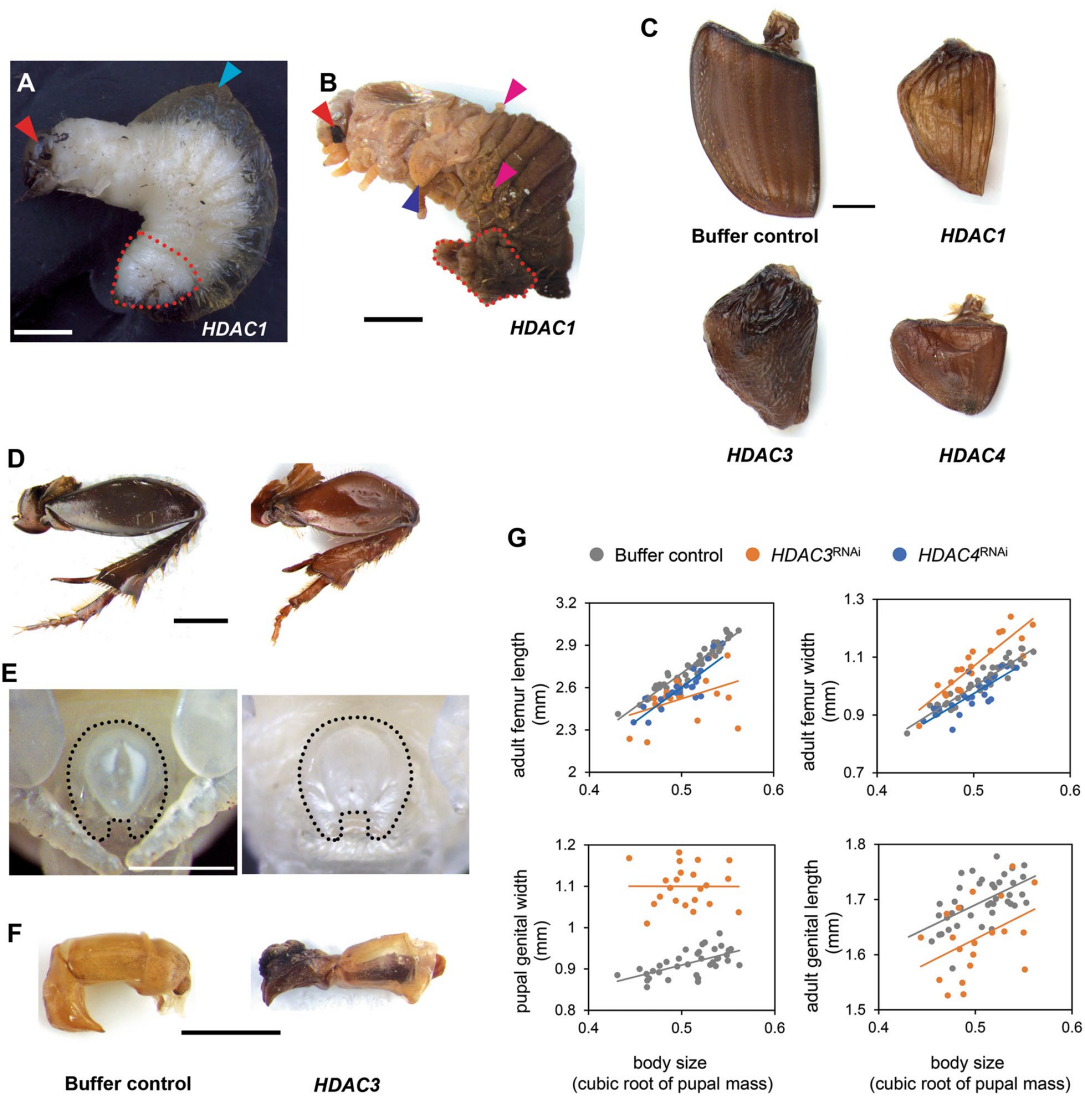
*HDAC*^RNAi^ effects on molting and appendage development.** A** and **B** Larval–pupal intermediate induced by *HDAC1* knockdown. The fat body accumulated outside of newly formed pupa is outlined (red dotted line) before (**A**) and after (**B**) peeling away the larval cuticle, respectively. Hemolymph in the cavity between larval and newly formed pupal cuticle, pupal compound eyes, wings, and pupal support structures are indicated by cyan, red, blue, and magenta arrowheads, respectively. **C** Representative wing phenotypes are shown as follows: buffer injection, *HDAC1*^RNAi^, *HDAC3*^RNAi^, and *HDAC4*^RNAi^. **D**-**F** Morphology of the hind leg (**D**), pupal genitalia (**E**), and adult aedeagus (**F**) compared to buffer-injected (left column) and *HDAC3*^RNAi^ (right column) individuals, respectively. **G** Knockdown of *HDAC3* (orange dots) or *HDAC4* (blue dots) on femur length and width compared to buffer-injected control (gray dots). Effects of *HDAC3*^RNAi^ on the male pupal and adult reproductive organ (bottom row in **G**). The RNAi phenotypes and their corresponding negative controls are shown at the same magnification. Scale bars: 1 mm

RNAi-mediated knockdown of *HDAC1* resulted in 100% larval mortality at the initial 1 μg/μl dsRNA injection dosage, as well as subsequent dosages as low as 0.25 μg/μl (Additional file 1: Table S1). Most individuals exhibited molting defects at the prepupal stages and eventually died with pupal traits, such as compound eyes, observed underneath the larval integument (Fig. 2A). Pupa-specific features, such as wings and pupal support structures, became visible when the larval cuticle was carefully removed (Fig. 2B). A mass of fat body accumulated underneath the posterior region of the developing pupal abdomen, resulting in a cavity filled with hemolymph between the larval integument and newly formed pupal cuticle (Fig. 2A). When dsRNA concentration was decreased to 0.01 μg/μl, very few individuals succeeded to develop to pupal (3/44) and adult (2/44) stages (Additional file 1: Table S1) amenable to phenotyping. In marked contrast, RNAi targeting *HDAC 3*, *4*, *6*, or *11* resulted in mortalities ranging from 16.7% to 86.7% depending on dosage and permitted more nuanced and quantitative analysis of phenotypic effects (Additional file 1: Table S1). Because no observable phenotypes were found following *HDAC6*^RNAi^ or *HDAC11*^RNAi^ even at high dsRNA dosage, we focus on *HDAC 1*, *3*, and *4* in the remainder.

### *HDACs* function during appendage development

To determine affected traits, we measured the scaling relationship of each trait to the cubic root of pupal mass as a proxy of body size since the more commonly used measure of thorax width was affected by *HDAC3* knockdown (treatment: *P* < 0.001, Additional file 1: Table S2). *HDAC1*^RNAi^ resulted in curtailment of both forewings (i.e., elytra) and hindwings at the pupal stage, which was retained into the adult (Fig. 2C). Similarly, both *HDAC3* or *HDAC4* knockdown diminished wing size (Fig. 2C). Thus, *HDAC 1, 3,* and* 4* appear to regulate wing development in similar ways. RNAi targeting *HDAC3* and *HDAC4* also affected leg development, but specific effects diverged. To assess leg phenotypes quantitatively we selected the femur, which is especially amenable to width and length measurement, for morphometric analyses. *HDAC4*^RNAi^ led to a reduction in femur length (treatment: *P* < 0.001, Additional file 1: Table S2) while the slope of the body size/femur length allometry was decreased in *HDAC3*^RNAi^ animals (treatment: *P* = 0.163; treatment × body size: *P* = 0.004, Fig. 2D and G, and Additional file 1: Table S2). In contrast, whereas *HDAC3*^RNAi^ increased femur width compared to control individuals (treatment: *P* < 0.001, Additional file 1: Table S2), the same measure was reduced in *HDAC4* knockdown animals (treatment: *P* = 0.001, Fig. 2D and G, and Additional file 1: Table S2). Lastly, we found that knockdown of *HDAC3* also affected the development of the male reproductive organ, the aedeagus, itself composed of the more proximal phallobase and the more distal parameres. Specifically, genital width increased and attained a more rounded shape at the pupal stage (treatment: *P* < 0.001, Fig. 2E and G, and Additional file 1: Table S2), while adult genitalia exhibited a deformation and overall shortening of both parameres and phallobase, which combined yielded a shortening of overall aedeagus length across all body sizes (treatment: *P* < 0.001, Fig. 2F and G, and Additional file 1: Table S2).

### *HDACs* function during thoracic and head horn formation

**Fig. 3:**
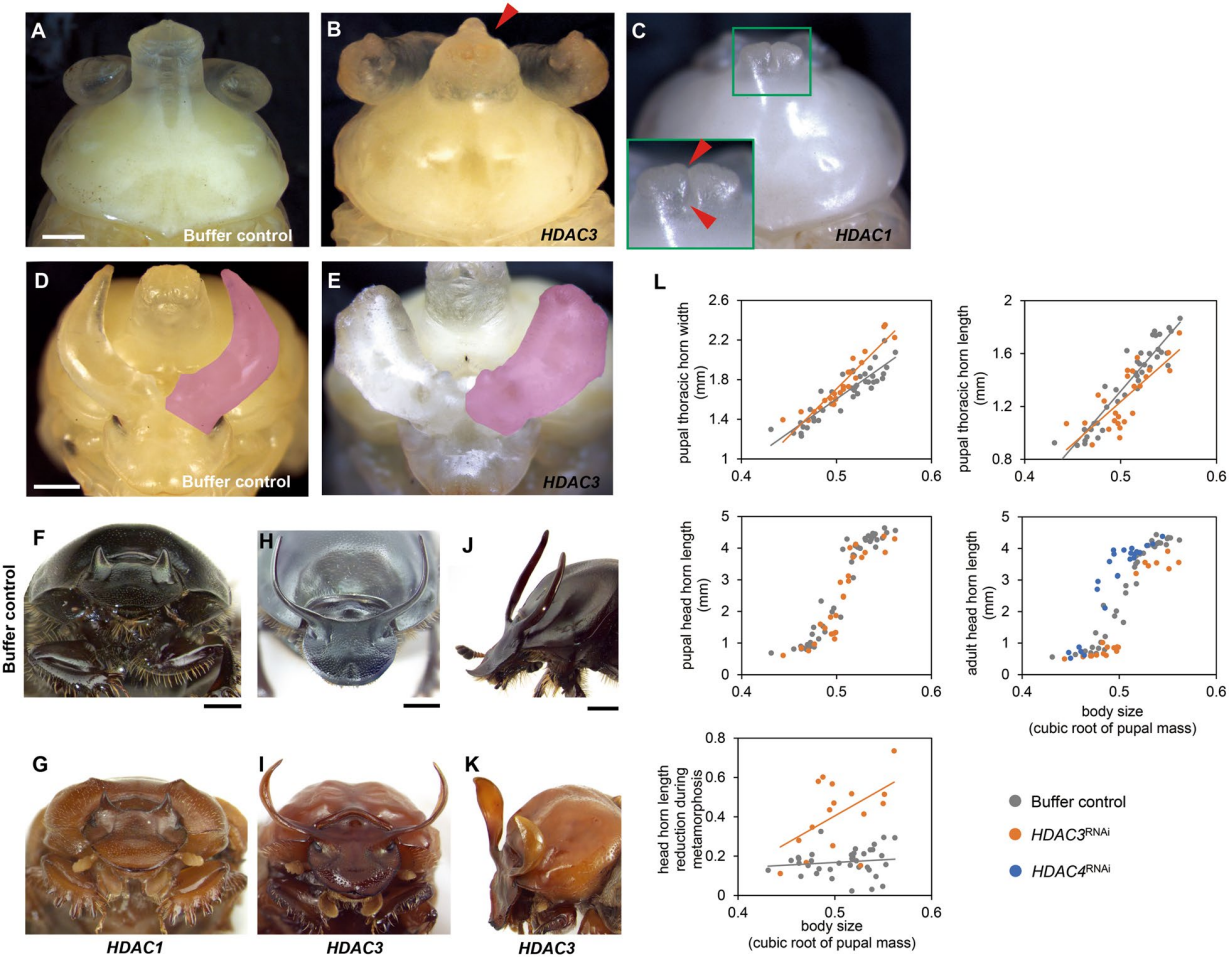
*HDAC*^RNAi^ effects on thoracic and head horn formation. **A**–**C** The pronotum in buffer-injected (**A**), *HDAC3*^RNAi^ (**B**), and *HDAC1*^RNAi^ (**C**) individuals. Inset in C shows the prothoracic horn. The furrow between paired horn vestiges is indicated by red arrowheads. **D** and **E** Representative head horn of negative control (**D**) and *HDAC3*^RNAi^ (**E**) individuals, respectively. The right head horn is colored magenta. **F**–**K** The morphology of the adult pronotum in front view (**F** and **G**), front view of head horns (**H** and **I**), as well as head horn viewed from lateral (**J** and **K**) in negative control (third row) individuals and following *HDAC* ^RNAi^ (bottom row), respectively. **L** Changes in thoracic and head horns resulting from *HDAC3*^RNAi^ and *HDAC4*^RNAi^. Reduction in head horn length during the transition from pupa to adult is increased by *HDAC3*^RNAi^ compared to buffer-injections (measured as the difference between pupal head horn length and adult head horn length in the same individual). RNAi phenotypes and their corresponding negative controls are shown at the same magnification. Scale bars: 1 mm

Head and thoracic horns are textbook examples of evolutionary novelties, and we sought to determine whether *HDAC* function may have been co-opted during the evolution of one or both horn types. *HDAC1*^RNAi^ resulted in a reduction in thoracic horn length and a split tip at the pupal stage (Fig. 3A and C), whereas the corresponding area in the adult exhibited a broad indentation (compared to the smoothly convex outline observed in wildtype or buffer control-injected individuals) and small bilateral projections at the respective edge of the indentation (Fig. 3F and G). Effects on head horns could not be quantified with certainty due to the high degree of natural variability of the trait and the very low number of surviving males, which in addition were too small to develop fully formed head horns. However, *HDAC3* knockdown caused measurable shape and scaling changes in thoracic horns. Specifically, *HDAC3*^RNAi^ increased pupal thoracic horn width (treatment: *P* < 0.001, Fig. 3A, B, L, and Additional file 1: Table S3), but decreased pupal thoracic horn length (treatment: *P* < 0.001, Fig. 3L and Additional file 1: Table S3). In addition, we found a bilateral indentation to the distal region of the thoracic horn, causing the thoracic horns of large *HDAC3*^RNAi^ pupae to attain a more conical shape (Fig. 3B). In contrast to thoracic horn phenotypes, *HDAC3*^RNAi^ yielded drastically enlarged head horns, in particular concerning head horn *width* across the entire range of male body sizes (Fig. 3D, E, H–K, and Additional file 1: Fig. S2). However, due to the highly varied nature of these phenotypes, we were unable to arrive at reliable landmarks for quantitative measure, hence this observation could only be made qualitatively. To determine whether head horn *length* was also affected, we further measured the scaling relationship of head horn length to body size. *HDAC3*^RNAi^ steepened the slope of the head horn length allometry at both pupal (treatment: *P* = 0.025, Fig. 3L and Additional file 1: Table S3) and adult stages (treatment: *P* = 0.012, Fig. 3L and Additional file 1: Table S3). Notably, *HDAC3*^RNAi^ also reduced the maximum asymptotic horn length in adults (treatment: *P* < 0.001, Fig. 3L and Additional file 1: Table S3), which was not detected at the pupal stage (treatment: *P* = 0.107, Fig. 3L and Additional file 1: Table S3). The quantification of head horn length reduction from pupa to adult further confirmed this observation (treatment: *P* < 0.05, Additional file 1: Table S2), and this effect was enhanced with increasing body size (treatment × body size effect: *P* = 0.010, Fig. 3L and Additional file 1: Table S2). These results suggest that *HDAC3*^RNAi^ affects head horn *width* during the horn growth phase (which takes place during the larval-to-pupal transition, thereby resulting in visible *pupal* phenotypes), but affects horn *length* during the pupal remodeling phase of ontogeny (thus becoming apparent in adults only). *HDAC4*^RNAi^ in turn not only decreased maximum asymptotic horn length in adults (treatment: *P* = 0.027, Additional file 1: Table S3), but also altered the body size threshold of sigmoidal allometry separating small hornless from large and fully horned males, causing relatively small males which would normally remain hornless to develop relatively large head horns instead (treatment: *P* < 0.001, Fig. 3L and Additional file 1: Table S3). In contrast, we did not find abnormal phenotypes with respect to thoracic horns in *HDAC4*^RNAi^ individuals.

## Discussion

### The significance of *HDACs* in horned beetle development and evolution

Earlier work on the broad-horned flour beetle *G. cornutus* was the first to document the role of *HDACs* in the regulation of nutrition-responsive plasticity in insects [21]. Large males in this species develop conspicuous mandibular projections (called mandibular horns) which were reduced following *HDAC1*^RNAi^, whereas *HDAC3*^RNAi^ led to hypertrophy. Opposite effects were observed with respect to wing size, yet none in genitalia [21]. These results were the first to suggest that *HDACs* operate in a trait-specific manner, and in particular contribute to the plastic and sex-specific expression of exaggerated male mandibles. Our results presented here further support a role of *HDACs* in the regulation of trait-specific plasticity, as well as add important new aspects to our understanding of HDAC function in insect development.

First, similar to the horns of rhinoceros beetle *Trypoxylus dichotomus*, *Onthophagus* horns frequently exhibit pronounced nutrition-dependent plasticity, in contrast to the more isometric growth typical of wings and legs, or the hypoallometric growth of genitalia [15, 32]. Several mechanisms have been proposed as possibly underlying module-specific conditional growth (e.g., insulin/insulin-like growth factor [14, 15], FOXO [16, 38, 39], HDAC [21]). Ozawa et al. [21], in particular, proposed a mechanistic explanation of epigenetic flexibility in which developmentally plastic organs (e.g., mandibular horns in *G. cornutus*) are more susceptible to epigenetic (i.e., *HDAC*) perturbation, whereas developmentally robust organs (e.g., genitalia) are non-responsive to *HDAC* perturbation. However, results presented here are at odds with this model. Specifically, even though head and thoracic horn development in *O. taurus* exhibit exaggerated nutritional plasticity, the effects of *HDAC3*^RNAi^ were considerably more pronounced in genitalia and wings. Genitalia in particular exhibited a considerable reduction of size relative to body size across all body sizes following *HDAC3*^RNAi^, thereby highlighting a previously unexpected role of *HDAC* in regulating the development of traits generally assumed to be robust to nutritional variation. Intriguingly, a similar outcome was observed following *insulin receptor* (*InR1*/*2*) transcript depletion in *O. taurus* [38]. Hence, the epigenetic flexibility hypothesis proposed in *Gnatocerus* beetles is unlikely to explain the findings seen here in *Onthophagus*, consistent with divergences in *HDAC* function across the Coleoptera, again similar to what has recently been reported for the insulin signaling pathway [40]. This in turn raises the possibility that, in addition to its primary function in regulating epigenetic status, *HDAC3* may also function in aspects of trait morphogenesis not related to nutritional status and developmental plasticity.

Second, we found that *HDAC1*^RNAi^ induced developmental arrest at the prepupal stages in line with previous studies in *Tribolium castaneum* [22], which suggests that *HDAC1* expression is required for suppressing the expression of genes involved in juvenile hormone (JH) action. In *Tribolium*, *HDAC1* knockdown prevents the larval-to-pupal transition via derepressing the expression of JH-response genes, thereby influencing JH actions and thus halting metamorphosis [22]. Similarly, severe *HDAC1* knockdown caused developmental failures during the pupal stage in *Gnatocerus*, indicating a possibly conserved role in basic developmental process mediating metamorphosis. However, despite this putative conservation of *HDAC1* function across the Coleoptera assessed to date, we also found that the precise nature and direction of *HDAC*^RNAi^ effects on appendage formation diverged between *Gnatocerus* and *Onthophagus* beetles even beyond those already noted above: for example, in *Onthophagus* downregulation of *HDAC1*, *3*, and *4* all appears to affect wing size similarly, whereas in *Gnatocerus HDAC1*^RNAi^ and *HDAC3*^RNAi^ yield opposite effects. Likewise, in *Onthophagus*, *HDAC3*^RNAi^ increased femur width, but *HDAC4*^RNAi^ decreased it, whereas leg morphology was generally unaffected in *Gnatocerus* beetle. Lastly, our results document the recruitment of *HDAC* function into the formation of an evolutionarily novel structure—head and thoracic horns—suggesting that *HDAC* function is not just evolutionarily labile among conserved insect traits but also contributed to the comparatively recent evolution of *Onthophagus* weaponry, including the regulation of size, shape, and key components of scaling.

### Development and evolution of pupal remodeling

The horns of adult beetles are the product of developmental processes operating at at least two distinct stages of development, a rapid growth phase approximately 48 h immediately prior to the larval-to-pupal molt and a remodeling phase during the pupal stage [33]. While generally given less attention, pupal remodeling can be quite extensive and fully formed pupal horns may be subject to considerable reduction and even complete resorption in many species. Thus, the morphological diversity of adult horns is not only influenced by the differential regulation of growth during the prepupal stage, but also by the developmental processes underlying the differential resorption of horn tissue during the pupal stage [33, 41, 42]. Previous work identified that differential programmed cell death facilitates species, sex, and body-region specific resorption of horn primordia [43]. However, the mechanisms regulating horn resorption during the pupal stage remain largely unknown. Our results implicate *HDAC3* as a regulator of both prepupal growth and pupal remodeling of horn primordia. Specifically, we show that *HDAC3*^RNAi^ increased head horn width, that this phenotype was already prominently visible at the pupal stage, and must therefore have resulted from modifications to the prepupal growth phase of horn formation (Fig. 3). In addition, however, we also find that *HDAC3*^RNAi^ altered horn *length* in a manner not evident at the pupal stage but clearly discernible in the resulting adults, and thus a consequence of *HDAC3*^RNAi^ effects on the pupal remodeling phase of horn formation. As such, *HDAC3* is one of relatively few genes identified to date to be involved in horn remodeling during the pupal stage of horned beetles [42, 44].

### Chromatin modifications and developmental plasticity

This work is the first to implicate chromatin modifications in the regulation of development and plasticity in horned dung beetles. While earlier work documented the existence of the complete methylation machinery in the *O. taurus* genome alongside sex- and nutrition-dependent differences in methylation signatures, the functional significance of chromatin modifications, if any, had remained unknown [45, 46]. We now show that downregulation of *HDAC3* and *HDAC4* affect critical aspects of horn formation including size, shape, and the location of the inflection point separating alternate male morphs. Future work will need to explore if and how *HDAC* functions may be contributing to the genome-wide remodeling patterns, and more generally the roles of *cis*-regulatory elements in the development and evolution of plasticity in insects.

## Methods

### Insects

Adult *O. taurus* were collected, courtesy of John Allen, from Paterson Farm near Ravenswood, Western Australia. A laboratory population was maintained at 25 ℃ in a sand/soil mixture and fed cow manure twice a week. Larvae used for injection were collected and prepared as described previously [47].

### Identification of *O. taurus* orthologs for HDACs

The *Onthophagus* orthologs of *HDAC* genes were identified via reciprocal BLAST to *T. castaneum*, *G. cornutus*, *Drosophila melanogaster*, *Bombyx mori*, *Apis mellifera*, and *Homo sapiens* in NCBI databases. Amino acid sequences of *HDAC* genes and *sirtuin-1*, a *NAD-dependent protein deacetylase* used as outgroup to the above species were aligned with MUSCLE algorithm implemented in MEGA X [48]. Neighbor-Joining tree (bootstrap replicates 1000) was constructed using MEGA X.

### Gene clone, double-stranded RNA (dsRNA) synthesis, and injection

To exclude potential off-target effect, we executed a bioinformatic search of selected gene regions for dsRNA design against the whole genome of *O. taurus* using the BlastN algorithm in NCBI, which enables sequence identity searches of a word-size down to seven bases, to ensure that no more than 20mers of identical sequences in loci other than the targeted genes existed within the genome. Total RNA was extracted with RNeasy Mini Plus Kit (QIAGEN) and reverse transcribed with iScript cDNA Synthesis kit (Bio-Rad). Partial fragments of each genes were amplified with PCR by using gene-specific primers (Additional file 1: Table S4) and cloned into pCR4-TOPO TA vector (Invitrogen, Thermo Fisher Scientific). After the sequences of the inserted gene fragment were confirmed by sequencing (Eurofins Genomics), DNA templates for in vitro transcription were produced with PCR by using TOPO RNAi primers (Additional file 1: Table S4) [49]. PCR products were purified and concentrated using the QIAquick PCR Purification Kit (QIAGEN) and subjected to in vitro transcription (MEGAscript T7 Transcription Kit, Thermo Fisher Scientific) and dsRNA purification (MEGAclear Transcription Clean-Up Kit, Thermo Fisher Scientific), according to the manufacturer’s protocol. DsRNA was quantified and stored at – 80 ℃ until use. Each individual was injected with 3 μl dsRNA at the early stage of the last larval instar (i.e., the third larval instar, L3). Past work showed that neither control injections with non-specific dsRNA derived from exogenous vectors nor buffer solution alone affect morphological trait formation including scaling in *Onthophagus* [18, 19, 50–53], and we therefore selected injections using buffer solution as a negative control treatment in this study. Control animals were injected with the same volume of injection buffer (1.4 mM NaCl, 0.07 mM Na_2_HPO_4_, 0.03 mM KH_2_PO_4_, and 4 mM KCl) and kept at the same condition as dsRNA injected animals (see Additional file 1: Table S1 for detailed information of injection).

### Effect of *HDAC3*- and *HDAC4*-RNAi on the scaling relationships between several morphological traits and body size

Since the usual measure of *Onthophagus* body size—thorax width (e.g., [38, 54])—was clearly affected by our RNAi treatments (Fig. 3B and Additional file 1: Table S2), we measured the cube root of pupal mass as a proxy for individual body size [55, 56]. We used t-tests to compare body sizes between each RNAi-treated and control group.

Consistent with previous studies, we analyzed nonlinear horn allometries using untransformed data (e.g., [38, 54]). We used the package drc [57] in R 3.5.2 [58] to fit the body size/head horn length distribution a four‐parameter log-logistic (Hill) function in the form:

$$y=c+ \frac{d-c}{1+{\text{exp}}(b({\text{log}}\left(x\right)-{\text{log}}(a))},$$

With *x* = body size, *y* = head horn length, *a* = body size at the point of inflection of the sigmoid curve, *b* = slope of the curve, *c* = minimum and *d* = maximum asymptotic horn lengths [54]. We then inferred whether a complex model including a sigmoidal regression per treatment (i.e., control and RNAi treatments) fitted our data better than a simpler model with one sigmoidal regression including the whole sample by means of the Akaike Information Criterion (AIC) [59]. The AIC measures relative model fit—the lower its value, the better the model fits to the experimental data [60]. Upon finding the complex model more fitting, we used Welch’s t-tests (with Holm–Bonferroni sequential correction where applicable) to compare parameter means (*a*, *b*, *c*, *d*) between control-injected and RNAi treatment groups [38, 61]. We compared control individuals to *HDAC3*^RNAi^ and *HDAC4*^RNAi^ individuals in the case of the adult horn allometry. As for pupal horn allometry, we compared *HDAC3*^RNAi^ to control individuals.

To inspect the effect of RNAi manipulations on the linear allometries of all the other morphological traits considered, we used the ANCOVA procedure implemented in SPSS Statistics 25 [62] to model trait size as a function of body size, treatment (*HDAC3*^RNAi^ or *HDAC4*^RNAi^ vs control-injected) and their interaction. Interactions were removed if non-significant. Data were log-transformed prior to analyses [63]. The reduction in horn size during metamorphosis (defined as pupal horn length—adult horn length) was analyzed similarly, using untransformed data as in other analyses of horn morphology. Statistics for morphometric analyses are provided in Additional file 1: Tables S2 and S3.

### Image processing

All images were captured with a digital camera (Scion) mounted to a dissecting microscope (Leica MZ16, Germany). Brightness and contrast of images were adjusted across the entire image with Adobe Photoshop CC 2017 (Adobe, USA).

### Supplementary Information

**Additional file 1: Fig. S1.** Phylogenetic analysis of HDAC orthologues. **Fig. S2.** *HDAC3*^RNAi^ head horn phenotypes across the range of male body sizes. **Table S1**. Injection information for this study. **Table S2. **Effects of *HDAC3* and *HDAC4* knockdowns on trait sizes. **Table S3. **Effects of *HDAC3* and *HDAC4* knockdowns on pupal and adult head horns. **Table S4. **The primer information used in this study.

## Data Availability

All data are available in the main text and Additional file 1.
