# Supplementary material for: Histone deacetylases regulate organ-specific growth in a horned beetle
Source: EvoDevo. 2024 Apr 5;15:4. doi: 10.1186/s13227-024-00223-5 (PMC10996171; doi:10.1186/s13227-024-00223-5)
Supplement: Supplementary file 1 — Additional file 1: Fig. S1. Phylogenetic analysis of HDAC orthologues. Fig. S2. HDAC3RNAi head horn phenotypes across the range of male body sizes. Table S1. Injection information for this study. Table S2. Effects of HDAC3 and HDAC4 knockdowns on trait sizes. Table S3. Effects of HDAC3 and HDAC4 knockdowns on pupal and adult head horns. Table S4. The primer information used in this study. [file 13227_2024_223_MOESM1_ESM.docx]

Supplementary Materials for

**Histone deacetylases regulate organ-specific growth in a horned beetle**

Yonggang Hu*, Jordan R. Crabtree, Anna L. M. Macagno, Armin P. Moczek

**This file includes:**

Figs. S1 to S2

Tables S1 to S4

**
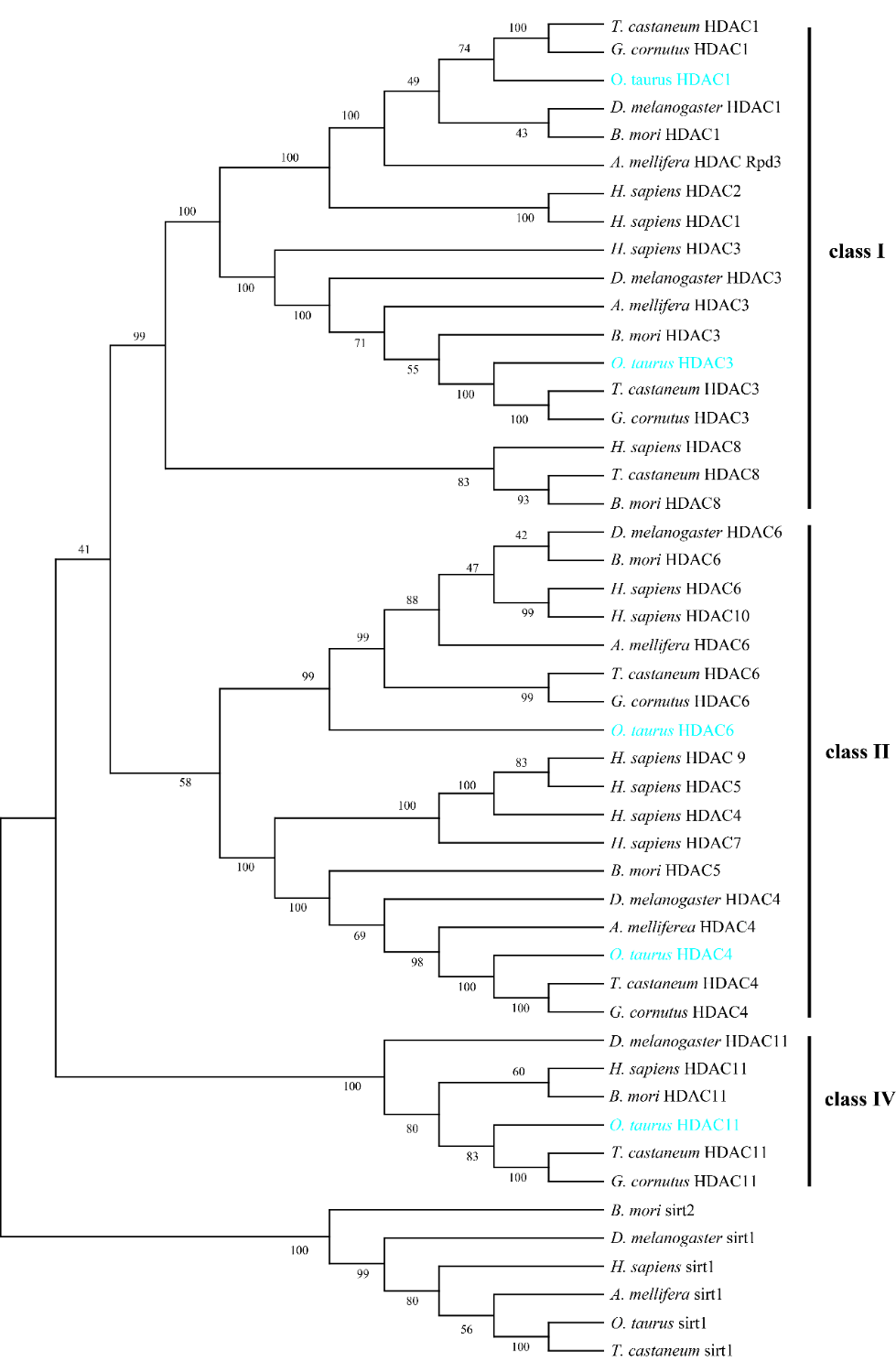
**

**Fig. S1. Phylogenetic analysis of HDAC orthologues**

Protein sequences of HDACs were aligned and then subjected to construction of Neighbor-joining phylogenetic tree. The NCBI accession numbers of protein sequences are listed as follows: *T. castaneum* HDAC1 (XP_966633.1), *G. cornutus* HDAC1 (BAW19561.1), *O. taurus* HDAC1 (XP_022902140.1), *D. melanogaster* HDAC1 (NP_647918.2), *H. sapiens* HDAC1 (BAG70111.1), *H. sapiens* HDAC2 (NP_001518.3), *T. castaneum* HDAC3 (XP_969419.1), *G. cornutus* HDAC3 (BAW19562.1), *O. taurus* HDAC3 (XP_022899704.1), *D. melanogaster* HDAC3 (NP_651978.2), *H. sapiens* HDAC3 (AAC52038.1), *H. sapiens* HDAC8 (AAF73428.1), *T. castaneum* HDAC8 (XP_008192051.1), *T. castaneum* HDAC6 (EFA11522.2), *G. cornutus* HDAC6 (BAW19564.1), *D. melanogaster* HDAC6 (ACN94041.1), *H. sapiens* HDAC6 (AAD29048.1), *O. taurus* HDAC6 (XP_022914414.1), *G. cornutus* HDAC4 (BAW19563.1), *T. castaneum* HDAC4/7 (XP_967425.2), *O. taurus* HDAC4 (XP_022905538.1), *D. melanogaster* HDAC4 (NP_001259507.1), *H. sapiens* HDAC7 (CAB55935.1), *H. sapiens* HDAC9 (AAO27363.1), *H. sapiens* HDAC4 (NP_001365345.1), *H. sapiens* HDAC5 (KAI2583350.1), *H. sapiens* HDAC11 (NP_079103.2), *D. melanogaster* HDAC11 (NP_001247296.1), *O. taurus* HDAC11 (XP_022906835.1), *G. cornutus* HDAC11 (BAW19565.1), *T. castaneum* HDAC11 (XP_970656.1), *B. mori* HDAC1 (XP_004931440.3), *B. mori* HDAC3 (XP_012552478.1), *B. mori* HDAC8 (XP_037872417.1), *B. mori* HDAC5 (XP_037868377.1), *B. mori* HDAC6 (XP_012543899.2), *B. mori* HDAC11 (XP_004925422.1), *A. mellifera* HDAC4 (XP_006570507.1), *A. mellifera* HDAC6 (KAG6801318.1), *A. mellifera* HDAC Rpd3 (XP_394976.4), *A. mellifera* HDAC3 (XP_395811.2), *T. castaneum* sirt1 (XP_008191290.1), *O. taurus* sirt1 (XP_022906438.1), *A. mellifera* sirt1 (XP_395386.3), *D. melanogaster* sirt1 (NP_477351.1), *H. sapiens* sirt1 (AAH12499.1), *B. mori* sirt2 (NP_001036937.1).

**
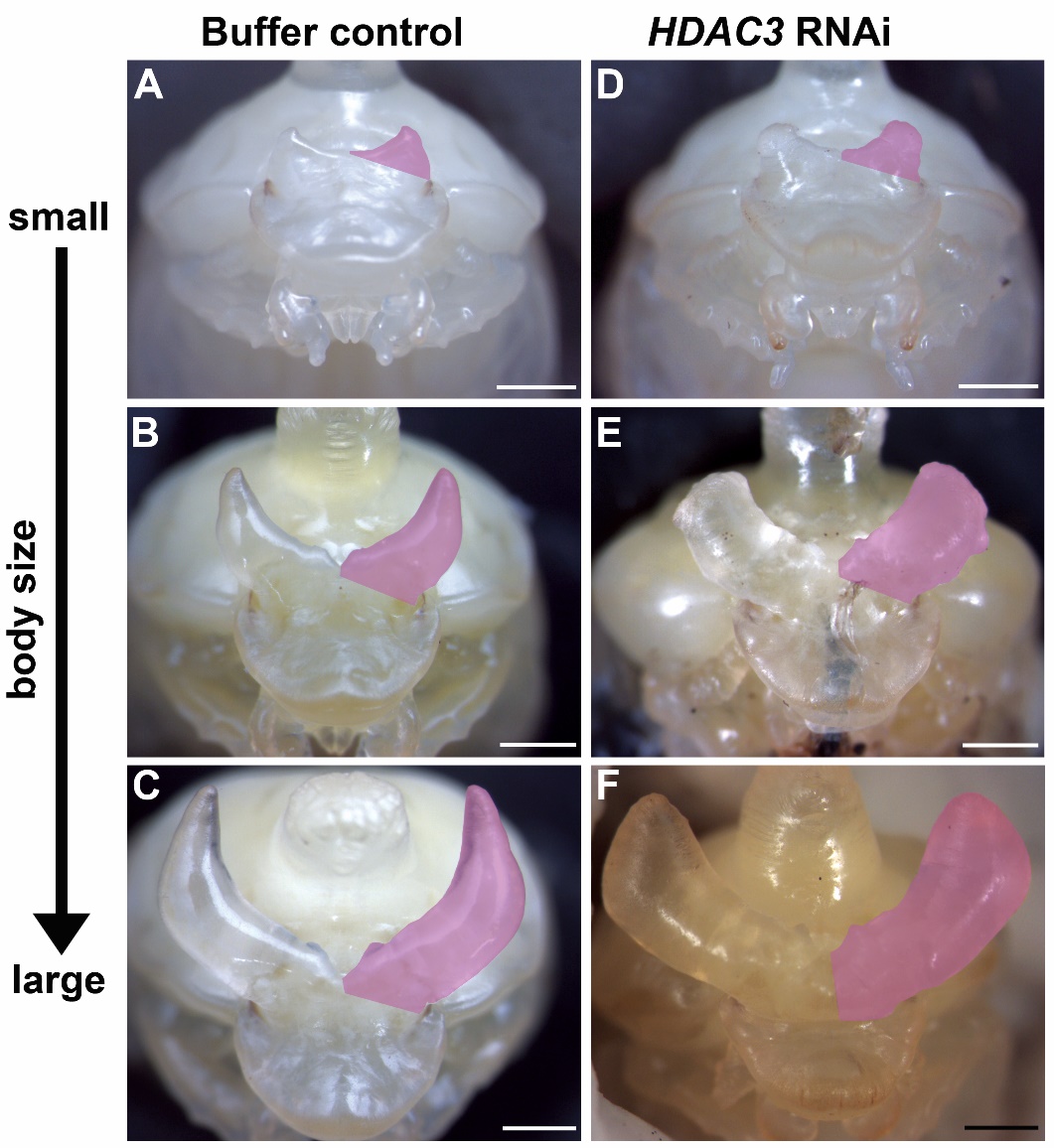
**

**Fig. S2. *HDAC3*^RNAi^ head horn phenotypes across the range of male body sizes**

(**A**-**F**) Dorsal view of the pupal head in buffer injected control individuals (left column) compared to *HDAC3*^RNAi^ individuals (right column) in (from top to bottom) small, medium-sized and large individuals, respectively. The left head horn along anterior/posterior axis is colored magenta. Scale bar: 1 mm.

**Table S1. Injection information for this study**

| **dsRNA** | **concentration (****μg/μl)** | **number of injected animals** | **survived to Pupa** | **survived to adult** | **mortality (died before eclosion, except technical lethality)** | **phenotypic penetrance** |
| --- | --- | --- | --- | --- | --- | --- |
| ***HDAC1*** | 1.0 | 25 | 0 | 0 | 100% | 100% |
|  | 0.5 | 22 | 0 | 0 | 100% | 100% |
|  | 0.25 | 53 | 0 | 0 | 100% | 100% |
|  | 0.1 | 71 | 1 | 0 | 100% | 100% |
|  | 0.05 | 54 | 4 | 3 | 94.4% | 50% |
|  | 0.01 | 44 | 3 | 2 | 95.5% | 66.6% |
| ***HDAC3*** | 1.0 | 60 | 12 | 8 | 86.7% | 100% |
|  | 0.5 | 176 | 43 | 29 | 83.4% | 95.3% |
| ***HDAC4*** | 1.0 | 18 | 7 | 7 | 61.1% | 100% |
|  | 0.5 | 62 | 39 | 35 | 43.5% | 89.7% |
| ***HDAC6*** | 2.0 | 23 | 20 | 19 | 17.4% | 17.4% |
|  | 1.0 | 18 | 16 | 15 | 16.7% | 16.7% |
| ***HDAC11*** | 2.0 | 18 | 11 | 11 | 35.3% | 35.3% |
|  | 1.0 | 30 | 25 | 24 | 17.2% | 17.2% |
| **buffer** | N/A | 80 | 74 | 73 | 1.4% | 1.4% |

Number of injected animals, the total number of injected larvae from at least two independent injections. Technical lethality, expected phenotype not recognized owing to lethality of the animals for example, by injection. In this study we defined the death of animals within 3 days after injection as technical lethality. Each individual represents one biological replicate.

**Table S2. Effects of *HDAC3* and *HDAC4* knockdowns on trait sizes**

|  | Type III Sum of Squares | df | Mean Square | *F* | *P* |
| --- | --- | --- | --- | --- | --- |
| Effect of *HDAC3*-RNAi on thoracic width | | | | | |
| Corrected Model | 0.202^a^ | 2 | 0.101 | 111.240 | 0.000 |
| Intercept | 13.652 | 1 | 13.652 | 15059.789 | 0.000 |
| Treatment | 0.017 | 1 | 0.017 | 18.594 | 0.000 |
| ln_curtPupaMass | 0.191 | 1 | 0.191 | 210.328 | 0.000 |
| Error | 0.056 | 62 | 0.001 |  |  |
| Total | 485.845 | 65 |  |  |  |
| Corrected Total | 0.258 | 64 |  |  |  |
| a. R^2^ = 0.782 (Adjusted R^2^ = 0.775) | | | | | |
| Effect of *HDAC3*-RNAi on thoracic length | | | | | |
| Corrected Model | 0.599^a^ | 2 | 0.300 | 502.653 | 0.000 |
| Intercept | 10.053 | 1 | 10.053 | 16866.410 | 0.000 |
| Treatment | 0.089 | 1 | 0.089 | 148.607 | 0.000 |
| ln_curtPupaMass | 0.485 | 1 | 0.485 | 813.509 | 0.000 |
| Error | 0.037 | 62 | 0.001 |  |  |
| Total | 429.138 | 65 |  |  |  |
| Corrected Total | 0.636 | 64 |  |  |  |
| a. R^2^ = 0.942 (Adjusted R^2^ = 0.940) | | | | | |
| Effect of *HDAC3*-RNAi on adult femur length | | | | | |
| Corrected Model | 0.234^a^ | 3 | 0.078 | 65.738 | 0.000 |
| Intercept | 6.603 | 1 | 6.603 | 5555.831 | 0.000 |
| Treatment | 0.002 | 1 | 0.002 | 1.992 | 0.163 |
| ln_curtPupaMass | 0.091 | 1 | 0.091 | 76.322 | 0.000 |
| Treatment × ln_curtPupaMass | 0.011 | 1 | 0.011 | 9.003 | 0.004 |
| Error | 0.071 | 60 | 0.001 |  |  |
| Total | 251.072 | 64 |  |  |  |
| Corrected Total | 0.306 | 63 |  |  |  |
| a. R^2^ = 0.767 (Adjusted R^2^ = 0.755) | | | | | |
| Effect of *HDAC4*-RNAi on adult femur length | | | | | |
| Corrected Model | 0.221^a^ | 2 | 0.111 | 268.207 | 0.000 |
| Intercept | 6.694 | 1 | 6.694 | 16248.194 | 0.000 |
| Treatment | 0.022 | 1 | 0.022 | 53.876 | 0.000 |
| ln_curtPupaMass | 0.181 | 1 | 0.181 | 438.427 | 0.000 |
| Error | 0.027 | 65 | 0.000 |  |  |
| Total | 268.622 | 68 |  |  |  |
| Corrected Total | 0.248 | 67 |  |  |  |
| a. R^2^ = 0.892 (Adjusted R^2^ = 0.889) | | | | | |
| Effect of *HDAC3*-RNAi on adult femur width | | | | | |
| Corrected Model | 0.360^a^ | 2 | 0.180 | 205.228 | 0.000 |
| Intercept | 1.143 | 1 | 1.143 | 1301.267 | 0.000 |
| Treatment | 0.064 | 1 | 0.064 | 72.840 | 0.000 |
| ln_curtPupaMass | 0.318 | 1 | 0.318 | 361.927 | 0.000 |
| Error | 0.054 | 61 | 0.001 |  |  |
| Total | 68.016 | 64 |  |  |  |
| Corrected Total | 0.414 | 63 |  |  |  |
| a. R^2^ = 0.871 (Adjusted R^2^ = 0.866) | | | | | |
| Effect of *HDAC4*-RNAi on adult femur width | | | | | |
| Corrected Model | 0.279^a^ | 2 | 0.140 | 177.141 | 0.000 |
| Intercept | 1.017 | 1 | 1.017 | 1289.299 | 0.000 |
| Treatment | 0.009 | 1 | 0.009 | 11.774 | 0.001 |
| ln_curtPupaMass | 0.255 | 1 | 0.255 | 322.847 | 0.000 |
| Error | 0.051 | 65 | 0.001 |  |  |
| Total | 67.823 | 68 |  |  |  |
| Corrected Total | 0.331 | 67 |  |  |  |
| a. R^2^ = 0.845 (Adjusted R^2^ = 0.840) | | | | | |
| Effect of *HDAC3*-RNAi on pupal genital width | | | | | |
| Corrected Model | 0.471^a^ | 2 | 0.236 | 183.235 | 0.000 |
| Intercept | 1.876 | 1 | 1.876 | 1458.133 | 0.000 |
| Treatment | 0.468 | 1 | 0.468 | 364.017 | 0.000 |
| ln_curtPupaMass | 0.011 | 1 | 0.011 | 8.288 | 0.006 |
| Error | 0.071 | 55 | 0.001 |  |  |
| Total | 56.279 | 58 |  |  |  |
| Corrected Total | 0.542 | 57 |  |  |  |
| a. R^2^ = 0.870 (Adjusted R^2^ = 0.865) | | | | | |
| Effect of *HDAC3*-RNAi on adult genital length | | | | | |
| Corrected Model | 0.036^a^ | 2 | 0.018 | 23.366 | 0.000 |
| Intercept | 4.164 | 1 | 4.164 | 5446.965 | 0.000 |
| Treatment | 0.019 | 1 | 0.019 | 24.384 | 0.000 |
| ln_curtPupaMass | 0.014 | 1 | 0.014 | 18.354 | 0.000 |
| Error | 0.044 | 57 | 0.001 |  |  |
| Total | 137.709 | 60 |  |  |  |
| Corrected Total | 0.079 | 59 |  |  |  |
| a. R^2^ = 0.451 (Adjusted R^2^ = 0.431) | | | | | |
| Effect of *HDAC3*-RNAi on pupal thoracic length | | | | | |
| Corrected Model | 2.275^a^ | 2 | 1.138 | 120.467 | 0.000 |
| Intercept | 0.210 | 1 | 0.210 | 22.221 | 0.000 |
| Treatment | 0.060 | 1 | 0.060 | 6.307 | 0.015 |
| ln_curtPupaMass | 2.167 | 1 | 2.167 | 229.471 | 0.000 |
| Error | 0.586 | 62 | 0.009 |  |  |
| Total | 108.091 | 65 |  |  |  |
| Corrected Total | 2.861 | 64 |  |  |  |
| a. R^2^ = 0.795 (Adjusted R^2^ = 0.789) | | | | | |
| Effect of *HDAC3*-RNAi on pupal thoracic width | | | | | |
| Corrected Model | 1.271^a^ | 2 | 0.635 | 171.029 | 0.000 |
| Intercept | 1.460 | 1 | 1.460 | 393.029 | 0.000 |
| Treatment | 0.070 | 1 | 0.070 | 18.959 | 0.000 |
| ln_curtPupaMass | 1.230 | 1 | 1.230 | 331.062 | 0.000 |
| Error | 0.230 | 62 | 0.004 |  |  |
| Total | 151.295 | 65 |  |  |  |
| Corrected Total | 1.501 | 64 |  |  |  |
| a. R^2^ = 0.847 (Adjusted R^2^ = 0.842) | | | | | |
| Effect of *HDAC3*-RNAi on head horn reduction during metamorphosis | | | | | |
| Corrected Model | 0.796^a^ | 3 | 0.265 | 25.009 | 0.000 |
| Intercept | 0.043 | 1 | 0.043 | 4.095 | 0.048 |
| Treatment | 0.050 | 1 | 0.050 | 4.666 | 0.036 |
| curtPupaMass | 0.113 | 1 | 0.113 | 10.665 | 0.002 |
| Treatment × curtPupaMass | 0.077 | 1 | 0.077 | 7.236 | 0.010 |
| Error | 0.520 | 49 | 0.011 |  |  |
| Total | 4.461 | 53 |  |  |  |
| Corrected Total | 1.316 | 52 |  |  |  |
| a. R^2^ = 0.605 (Adjusted R^2^ = 0.581) | | | | | |

Trait size was modeled as a function of body size, treatment (*HDAC3*-RNAi or *HDAC4*-RNAi vs control-injected) and their interaction using general linear models. Models were simplified by removing non-significant interactions (*P* > 0.05).

**Table S3. Effects of *HDAC3* and *HDAC4* knockdowns on pupal and adult head horns**

| Effect of *HDAC3*-RNAi on pupal head horn | | | | | | | | | | | | | | | | |
| --- | --- | --- | --- | --- | --- | --- | --- | --- | --- | --- | --- | --- | --- | --- | --- | --- |
|  | Buffer injection | | | | *HDAC3* | | | | Buffer VS *HDAC3* | |  |  |  |  |  |  |
|  | estimate | SE | SD | N | estimate | SE | SD | N | *P* | |  |  |  |  |  |  |
| slope | -43.130 | 7.824 | 53.635 | 47 | -83.928 | 19.671 | 98.353 | 25 | 0.0254 | |  |  |  |  |  |  |
| lower | 0.748 | 0.177 | 1.210 | 47 | 0.982 | 0.165 | 0.823 | 25 | 0.39 | |  |  |  |  |  |  |
| upper | 4.506 | 0.143 | 0.979 | 47 | 4.128 | 0.169 | 0.845 | 25 | 0.107 | |  |  |  |  |  |  |
| inflection | 0.501 | 0.002 | 0.016 | 47 | 0.506 | 0.002 | 0.008 | 25 | 0.1475 | |  |  |  |  |  |  |
| Effect of *HDAC3*- and *HDAC4*-RNAi on adult head horn | | | | | | | | | | | | | | | | |
|  | Buffer | | | | *HDAC4* | | | | *HDAC3* | | | | Buffer VS *HDAC4* | | Buffer VS *HDAC3* | |
|  | estimate | SE | SD | N | estimate | SE | SD | N | estimate | SE | SD | N | *P* | | *P* | |
| slope | -44.315 | 6.865 | 44.488 | 42 | -49.251 | 11.971 | 59.855 | 25 | -118.660 | 39.519 | 176.734 | 20 | 0.701 | | 0.0123 | |
| lower | 0.656 | 0.139 | 0.901 | 42 | 0.254 | 0.319 | 1.597 | 25 | 0.665 | 0.102 | 0.454 | 20 | 0.1915 | | 0.9666 | |
| upper | 4.403 | 0.128 | 0.827 | 42 | 3.971 | 0.123 | 0.616 | 25 | 3.578 | 0.127 | 0.568 | 20 | 0.027 | | 0.0002 | |
| inflection | 0.505 | 0.002 | 0.012 | 42 | 0.475 | 0.003 | 0.013 | 25 | 0.509 | 0.003 | 0.013 | 20 | 0.0001 | | 0.237 | |

Model fitted: Log-logistic (ED50 as parameter) (4 parms). Slope = slope of each sigmoid (b), lower = minimum asymptotic horn length (c), upper = maximum asymptotic horn length (d), inflection = body size at the inflection point (a). During the pupal stage, some injected individuals may die, or the horn might be affected artificially during phenotypic observation. Hence, the number of adult samples suitable for morphometric analyses is often less than that of pupa.

**Table S4. The primer information used in this study**

| **name** | **primer F** | **primer R** | **GenBank Accession No.** | **length (bp)** | **purpose** |
| --- | --- | --- | --- | --- | --- |
| *HDAC1* | CGTCAAACGTTACGGGCTTC | GCGATTGTCCCTCCTTCCAC | XM_023046372.1 | 445 | gene clone |
| *HDAC3* | TGTTTTGCAATGTGGGGCTG | GAATCGCGTCTGGTGGGATA | XM_023043936.1 | 401 | gene clone |
| *HDAC4* | ACCTTGCGGAGGAATTGGAG | CGAATCCAGCGGAAACCAAC | XM_023049770.1 | 554 | gene clone |
| *HDAC6* | GTTGGATGATGCACCAGGGA | AATCATAGCCACACCCGCTC | XM_023058646.1 | 643 | gene clone |
| *HDAC11* | CAACGATGAAACGGTTACAAC | AGTGATGTCAGCATAAGGGCA | XM_023051067.1 | 313 | gene clone |
| TOPO RNAi Primer | TAATACGACTCACTATAGGGCGAATTCGCCCTT | TAATACGACTCACTATAGGGCGAATTCGCCCTT | N/A | N/A | DNA template for *in vitro* transcription |
